# Development of a Highly Efficient Hybrid Peptide That Increases Immunomodulatory Activity Via the TLR4-Mediated Nuclear Factor-κB Signaling Pathway

**DOI:** 10.3390/ijms20246161

**Published:** 2019-12-06

**Authors:** Lulu Zhang, Xubiao Wei, Rijun Zhang, Matthew Koci, Dayong Si, Baseer Ahmad, Junhao Cheng, Junyong Wang

**Affiliations:** 1 Laboratory of Feed Biotechnology, State Key Laboratory of Animal Nutrition, College of Animal Science and Technology, China Agricultural University, Beijing 100193, ChinaWeixubiao01@126.com (X.W.); lionsdy1@gmail.com (D.S.); dr.baseerahmadkhan@gmail.com (B.A.); chengjunhao@cau.edu.cn (J.C.);; 2 Prestage Department of Poultry Science, North Carolina State University, Raleigh, NC 27695, USA; mdkoci@ncsu.edu

**Keywords:** immunoregulatory activity, cyclophosphamide, TLR4/MD-2, NF-κB

## Abstract

Immunity is a defensive response that fights disease by identifying and destroying harmful substances or microbiological toxins. Several factors, including work-related stress, pollution, and immunosuppressive agents, contribute to low immunity and poor health. Native peptides, a new class of immunoregulatory agents, have the potential for treating immunodeficiencies, malignancies, and infections. However, the potential cytotoxicity and low immunoregulatory activity and stability of native peptides have prevented their development. Therefore, we designed three hybrid peptides (LTA_a_, LTA_b_, and LTA_c_) by combining a characteristic fragment of LL-37 with an active Tα1 center that included Tα1 (17–24), Tα1 (20–25), and Tα1 (20–27). The best hybrid peptide (LTA_a_), according to molecule docking and in vitro experiments, had improved immunoregulatory activity and stability with minimal cytotoxicity. We investigated the immunoregulatory effects and mechanisms of LTA_a_ using a cyclophosphamide-immunosuppressed murine model. LTA_a_ effectively reversed immunosuppression by enhancing immune organ development, activating peritoneal macrophage phagocytosis, regulating T lymphocyte subsets, and increasing cytokine (tumor necrosis factor-alpha, interleukin-6, and interleukin-1β) and immunoglobulin (IgA, IgG, and IgM) contents. The immunomodulatory effects of LTA_a_ may be associated with binding to the TLR4/MD-2 complex and activation of the NF-κB signaling pathway. Therefore, LTA_a_ could be an effective therapeutic agent for improving immune function.

## 1. Introduction

Immunity is a defensive response than can protect against disease by identifying and destroying harmful substances or microbiological toxins [1]. A suppressed immune system can make an organism more susceptible to infection, organ injury, and cancer [2], and immunity plays an important role in preventing and recovering from these immune-mediated diseases. Therefore, it is necessary to develop a new immunomodulatory agent that can prevent and treat the diseases associated with immunosuppression.

Toll-like receptors (TLRs) are one of the pattern recognition receptors for conserved molecular patterns on microbial pathogens, and play important roles in host defense and in regulating immune responses [3]. TLR4 was the first TLR characterized, and leads to the activation of an immune response [4,5]. MD2 is an accessory receptor lacking a transmembrane domain, and forms a complex with TLR4 to produce a signaling-competent receptor [6]. The dimerization of TLR4/MD2 leads to the release of various proinflammatory cytokines such as tumor necrosis factor alpha (TNF-α), interleukin-6 (IL-6), and IL-1β [7]. These cytokines are also related to immune responses and have been implicated in host defense against pathogens [8]. Therefore, TLR4/MD2 ligands are promising candidates as vaccine adjuvants and pharmaceuticals that support immunotherapies.

Thymosin alpha-1 (Tα1) is a natural peptide with 28 amino acids and functions to enhance cell-mediated immunity [9]. Tα1 exerts its immunoregulatory activity by inducing CD4+ T cell activation and the humoral response [10,11]. In addition, Tα1 stimulates the production of various proinflammatory cytokines such as interferon-γ (IFN-γ), IFN-α, and interleukin-2 (IL-2) [12]. Due to immunoregulatory activity and low cytotoxicity of Tα1, it is used in the treatment of immunodeficiencies, malignancies, and infections [13,14,15].

LL-37 is a human cationic host-defense peptide that is a potent modulator of the innate immune response [16,17]. LL-37 can induce the production and release of various immunoregulatory mediators, proinflammatory cytokines, and chemokines [18,19,20]. Furthermore, recent studies have demonstrated that LL-37 could also modulate immunity by binding to the TLR-4/MD2 complex [21,22]. Therefore, LL-37 could prevent or attenuate immunosuppression.

Tα1 plays a vitally important role in the process of immune enhancement, but its subcutaneous half-life is very short, which decreases its efficacy and bioavailability. LL-37 has a long half-life, but its significant cytotoxicity has hindered its further development as a therapeutic drug [23]. Hybridization is a simple and effective strategy that combines the advantages of different native peptides, and has been proposed as a method of prolonging the half-lives, increasing the immunoregulatory activity, and reducing the undesirable cytotoxic effects of native peptides [24,25]. LL-37 (13-36) is a linear, cationic peptide that can effectively bind to the TLR4/MD2 complex and modulate immunity [21,22]. Tα1 (17–24), Tα1 (20–25), and Tα1 (20–27) also exhibit robust immunoregulatory activity [26,27]. We designed three hybrid peptides by combining a characteristic fragment of LL-37 with an active Tα1 center that included Tα1 (17-24) (Tα1a), Tα1 (20-25) (Tα1b), and Tα1 (20–27) (Tα1c). We hypothesized that hybridization would increase immunoregulatory activity, prolong the half-life, and decrease cytotoxicity. The best hybrid peptide, based on these criteria, was screened by molecule docking and in vitro experiments. The immunoregulatory mechanisms of LTA_a_ were further analyzed by exploring the molecular basis of its immunoregulatory effects using a cyclophosphamide (CTX)-immunosuppressed murine model.

## 2. Results

### 2.1. Selection of Immunomodulatory Peptides by Molecular Docking

**Figure 1:**
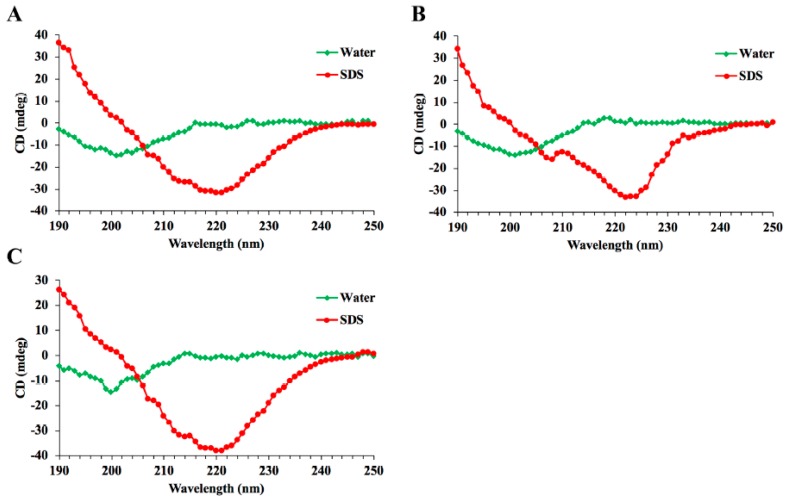
The circular dichroism (CD) spectra of the peptides. The peptides, including LTA_a_ (**A**), LTA_b_ (**B**), and LTA_c_ (**C**), were dissolved in sterile water and 25 mM sodium dodecyl sulfate (SDS). The CD spectra assays were performed over a UV range of 190–250 nm at 25 °C on the jasco-810 spectropolarimeter.

**Figure 2:**
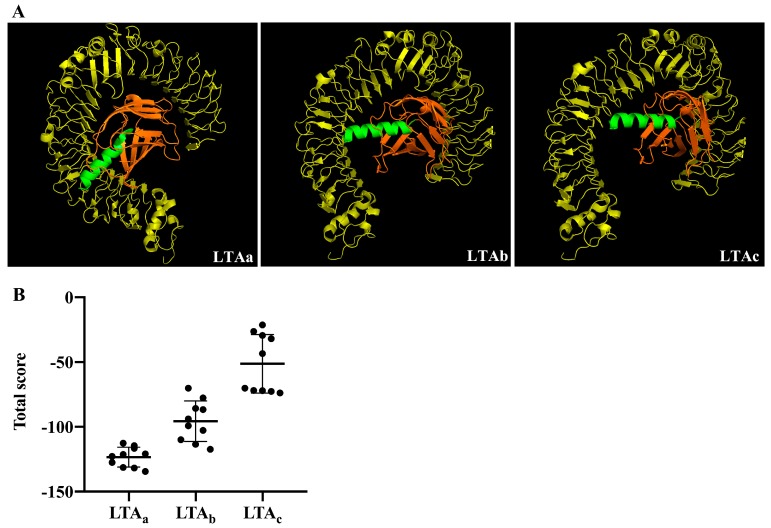
Overall structure of the hybrid peptide-TLR4/MD-2 complex. (**A**) Hybrid peptides binding to TLR4/MD-2. The yellow ribbons represent TLR4, the orange ribbons MD2, and the green ribbons hybrid peptides. (**B**) Energy plot of 10 of 100 decoy structures from an TLR4/MD-2 docking study by RosettaDock. Data are means ± Standard Error of Mean (SEMs).

The sequences of three hybrid peptides, LL-37-Tα1_a_ (LTA_a_), LL-37-Tα1_b_ (LTA_b_), and LL-37-Tα1c (LTA_c_), were shown in Table 1. The structure model of the peptides using PyMOL software showed that all the hybrid peptides adopt an α-helix structure, which was then verified by circular dichroism (CD) spectroscopy (Figure 1). The results showed that all the hybrid peptides adopt an α-helix structure in 25 mM sodium dodecyl sulfate (SDS) and a random coil structure in water. These data indicated all these three peptides are α-helix peptide. In order to evaluate the immunomodulatory activity of the three hybrid peptides, the binding modes of the hybrid peptides to TLR4-MD-2 were analyzed by molecular docking. LTA_a_ had more favorable docking scores to TLR4-MD-2 than the other hybrid peptides, and the total score was lower than −100 (Figure 2A,B).

**Figure 3:**
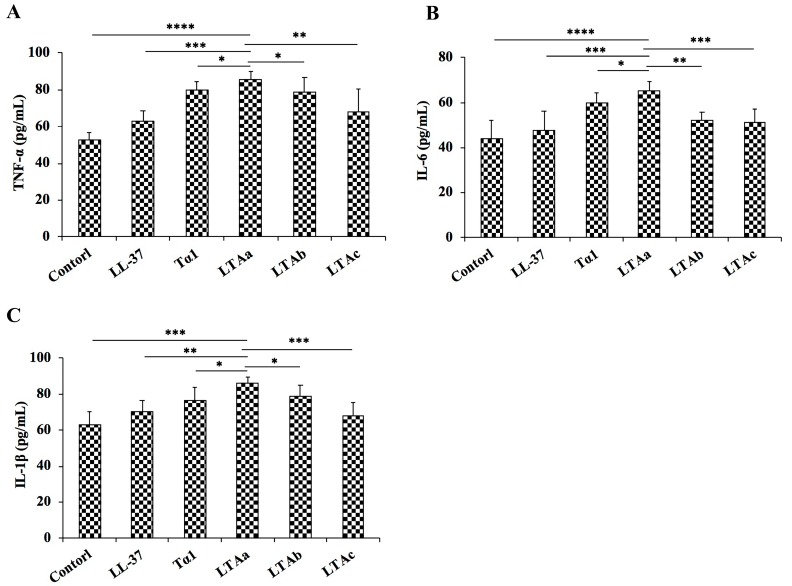
Effect of hybrid peptides on cytokine production. Cells were treated with 10 μg/mL peptides for 12 h, and protein levels of TNF-α (**A**), IL-6 (**B**), and IL-1β (**C**) were quantified by enzyme-linked immunosorbent assay. Data are means ± SEMs of five biological replicates. *, *p* ≤ 0.05; **, *p* ≤ 0.01; ***, *p* ≤ 0.001; and ****, *p* ≤ 0.0001.

After the molecular docking assay, we evaluated the immunomodulatory activity of the peptides in vitro using mouse macrophages (RAW264.7). As shown in Figure 3, all of the parental peptides and hybrid peptides increased TNF-α (Figure 3A), IL-6 (Figure 3B), and IL-1β (Figure 3C) production. The 10 μg/mL dose of LTA_a_ caused remarkable increases in the secretion of TNF-α, IL-6, and IL-1β in comparison with the other peptides tested. Therefore, LTA_a_ was selected for further immunomodulatory experiments.

### 2.2. Cytotoxicity to RAW264.7 Macrophage Cells

**Figure 4:**
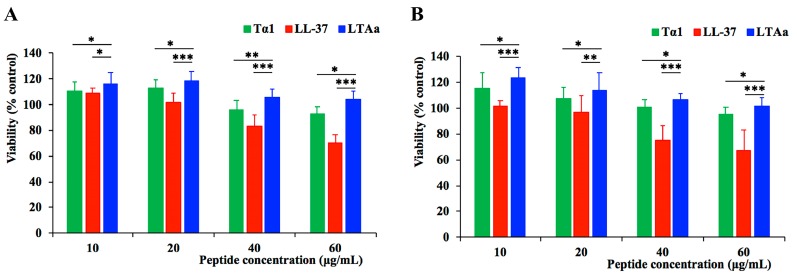
Effect of LTA_a_ on RAW264.7 cell viability as determined by a Cell Counting Kit-8 (CCK-8) assay. RAW264.7 viability was measured using a CCK-8 assay kit. Briefly, cells were plated at a density of 1.0–2.0 × 104 cells per well and then treated with peptides (10–60 μg/mL) or without peptides. After incubation for 24 h (**A**) or 72 h (**B**) at 37 °C, the cells were incubated with CCK-8 solution for 4 h at 37 °C in the dark. The optical density was measured using a microplate reader at 450 nm. Data are means ± SEMs of eight biological replicates. *, *p* ≤ 0.05; **, *p* ≤ 0.01; and ***, *p* ≤ 0.001.

The cytotoxicity of LTA_a_ and its parental peptides was evaluated by conducting a CCK-8 assay using RAW264.7 macrophage cells (Figure 4). Even at the highest concentration of 60 μg/mL, LTA_a_ exhibited no significant cytotoxicity and had higher cell survival rates than its parental peptides LL-37 and Tα1, both after 24 h (Figure 4A) and 72 h (Figure 4B).

### 2.3. Ex Vivo Stability of LTA_a_ in Plasma

**Figure 5:**
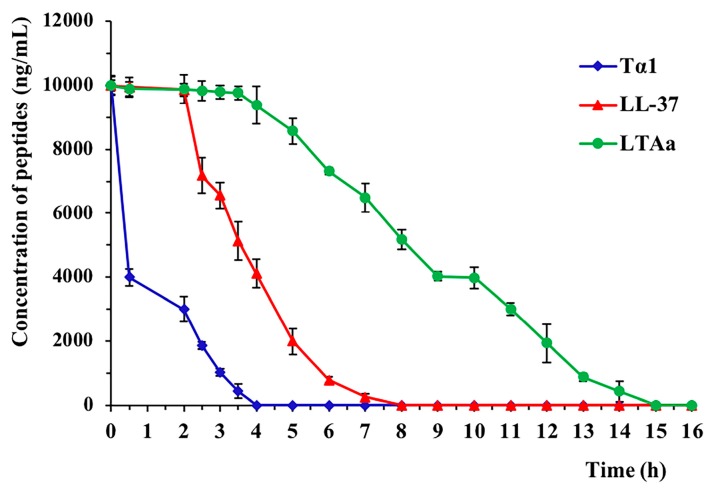
Mean plasma peptide concentrations over time. The plasma concentrations of LL-37, Tα1, and LTA_a_ in vitro were quantified by high-performance liquid chromatography. Data are means ± SEMs of three biological replicates.

The plasma concentration of each target peptide over time is shown in Figure 5. The half-life (t1/2) of Tα1 was less than 2 h, which is consistent with previous reports [9,28]. LTA_a_ had a significantly longer half-life than Tα1 or LL-37 in plasma (Table 2).

### 2.4. Effect of LTA_a_ on Body Weight and Immune Organs

**Figure 6:**
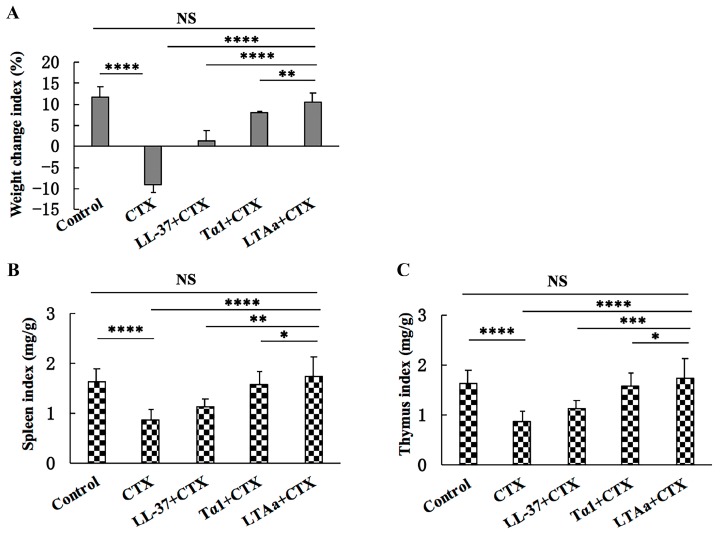
Protective effects of LTA_a_ on body weight (**A**), spleen index (**B**), and thymus index (**C**). The mice were randomly divided into five groups (12 mice in each group): a control group, a cyclophosphamide (CTX) group, a LL-37 group, a Tα1 group, and a LTA_a_ group. For the first 3 days, CTX (80 mg/kg mouse weight) was administered intraperitoneally once daily to establish the immunosuppressed animal model. From days 4 to 10 (7 days), peptides (10 mg/kg mouse weight) were administered intraperitoneally each day. The CTX group was only treated with CTX, and the control group was given sterile saline. The body weights of the mice were recorded before and after the experiment. The spleen weights and thymus weights of the mice were recorded before and after the experiment. Data are means ± SEMs of 12 biological replicates. Not significant (NS), *p* > 0.05; *, *p* ≤ 0.05; **, *p* ≤ 0.01; ***, *p* ≤ 0.001; and ****, *p* ≤ 0.0001.

As shown in Figure 6A, the body weights of immunosuppressed mice in the CTX group were significantly lower than those in the control group. After LTA_a_ treatment, the mice rapidly recovered their weight. Therefore, LTA_a_ appears to be more potent than the parental peptides.

As expected, the CTX mice had significantly lower spleen (Figure 6B) and thymus (Figure 6C) index values than the control mice, but LTA_a_ treatment reversed this effect. In addition, the spleen and thymus indices values in the LTA_a_-treated group were significantly higher than those in the parental peptides groups.

### 2.5. Effects of LTA_a_ on Peritoneal Macrophage Phagocytosis

**Figure 7:**
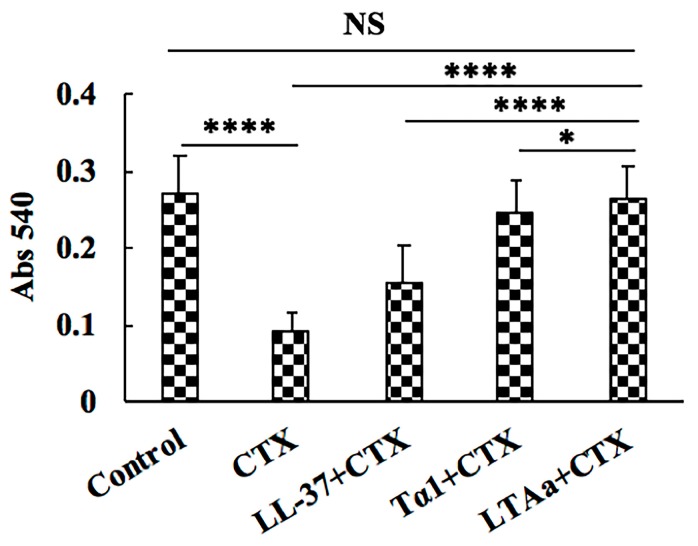
Effects of LTA_a_ on macrophage phagocytosis in mice immunosuppressed by cyclophosphamide (CTX). Peritoneal macrophages were aseptically obtained from the different groups of mice. The phagocytic capacity of the peritoneal macrophages was measured by the neutral red uptake method. A neutral red solution (50 mg/mL) was added to each well and incubated for an additional 3 h. The cells were then washed with phosphate buffer saline (PBS) three times and the phagocytotic dye was extracted with 100 μL 1% acetic acid solution (v/v) in 50% ethanol (v/v). The optical density (OD) was measured at 540 nm. Data are means ± SEMs of 12 biological replicates. Not significant (NS), *p* > 0.05; *, *p* ≤ 0.05; and ****, *p* ≤ 0.0001.

To investigate the effects of LTA_a_ on peritoneal macrophages in CTX-immunosuppressed mice, the macrophage phagocytic rate was calculated based on neutral red (0.75%) uptake. As shown in Figure 7, the phagocytic rate was significantly lower in the CTX-treated group (31.93%) than in the control group (55.67%). Mice in the LTA_a_-treated group had significantly higher phagocytic rates than those in the Tα1- or LL-37-treated groups.

### 2.6. Effects of LTA_a_ on T cells in Mice Splenocytes

**Figure 8:**
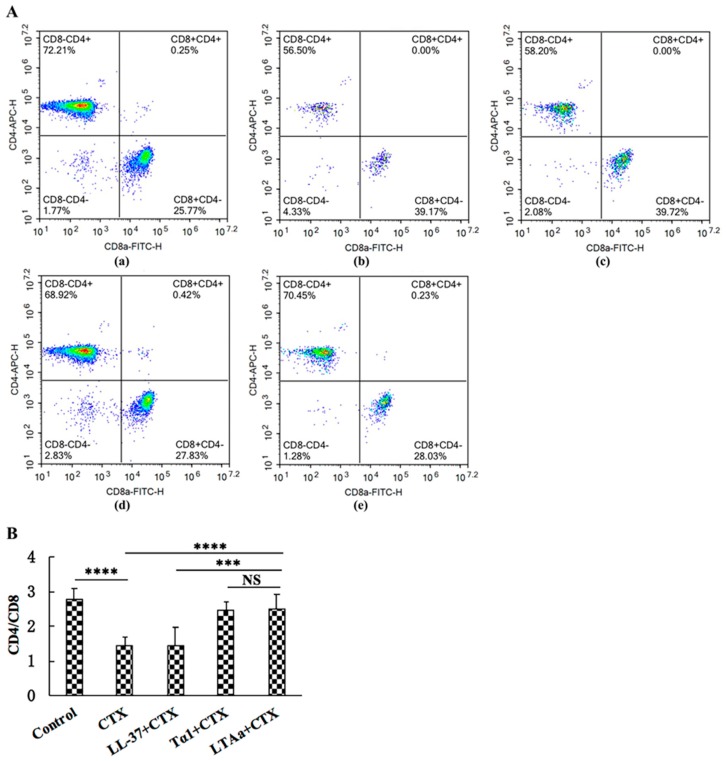
Effects of LTA_a_ on T lymphocyte subpopulations in splenocytes. The spleen was collected from the different groups of mice, ground, and harvest the cell suspension. The cells were stained with CD3- PerCP, CD4-APC, and CD8-FITC for 30 min at 4 °C for T lymphocyte measurements. (**A**) The percentage of different T cell subsets was analyzed by flow cytometry. (**A-a**) Control, (**A-b**) cyclophosphamide (CTX), (**A-c**) LL-37 + CTX, (**A-d**) Tα1 + CTX, (**A-e**) LTA_a_ + CTX. Bivariate plots are shown as representative, independent assessments that were quantified and plotted as the CD4^+^:CD8^+^ ratio in part (**B)**. Data are means ± SEMs of 12 biological replicates. Not significant (NS), *p* > 0.05; ***, *p* ≤ 0.001; and ****, *p* ≤ 0.0001.

To investigate the effects of LTA_a_ on cellular immunity, CD4^+^ and CD8^+^ T lymphocyte levels were determined by flow cytometry. The proportions of CD4^+^ and CD8^+^ T lymphocytes (Figure 8A,B) in the spleen were significantly lower in the CTX group than in the control group. The LTA_a_-treated group had higher CD4^+^:CD8^+^ values than the group treated with CTX alone. The LTA_a_-treated group had a significantly higher CD4^+^:CD8^+^ T lymphocyte ratio (*p* ≤ 0.05) than the LL-37 group. The Tα1- and LTA_a_-treated groups had similar CD4^+^:CD8^+^ T lymphocyte ratios (*p* > 0.05).

### 2.7. Effects of LTA_a_ on Serum TNF-α, IL-6, and IL-1β Levels

**Figure 9:**
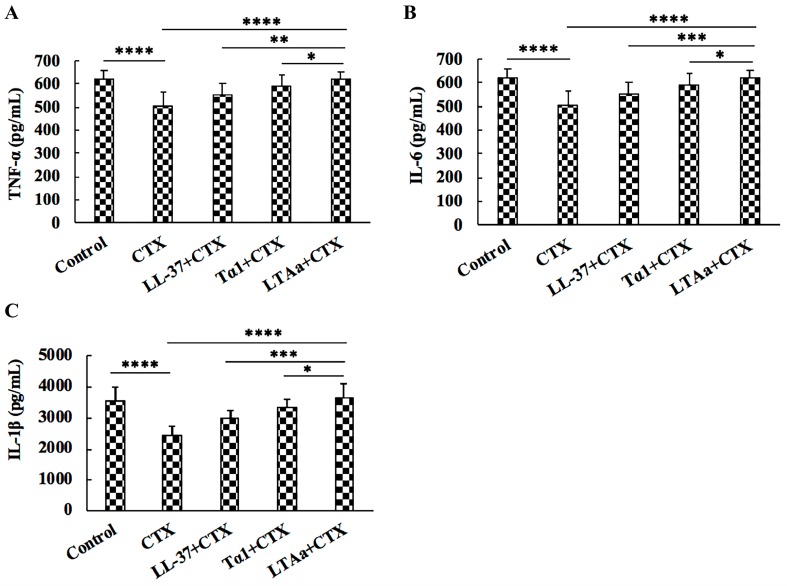
Effects of LTA_a_ on TNF-α **(A)**, IL-6 **(B)**, and IL-1β **(C)** levels in mice immunosuppressed by cyclophosphamide (CTX). Levels of TNF-α, IL-6 and IL-1β were detected in serum from different groups of mice through enzyme linked immunosorbent assay (ELISA). Data are means ± SEMs of 12 biological replicates. *, *p* ≤ 0.05; **, *p* ≤ 0.01; ***, *p* ≤ 0.001; and ****, *p* ≤ 0.0001.

To investigate the protective effects of LTA_a_ against immunosuppression in CTX-treated mice, TNF-α, IL-6, and IL-1β secretion was evaluated by ELISA. As shown in Figure 9, CTX injection caused significant reductions in TNF-α, IL-6, and IL-1β levels. All of the peptides tested reversed the declines in TNF-α (Figure 9A), IL-6 (Figure 9B), and IL-1β (Figure 9C). Mice in the LTA_a_-treated group had significantly higher TNF-α, IL-6, and IL-1β concentrations than those in the Tα1- or LL-37-treated groups.

### 2.8. Effects of LTA_a_ on Serum Ig Contents

**Figure 10:**
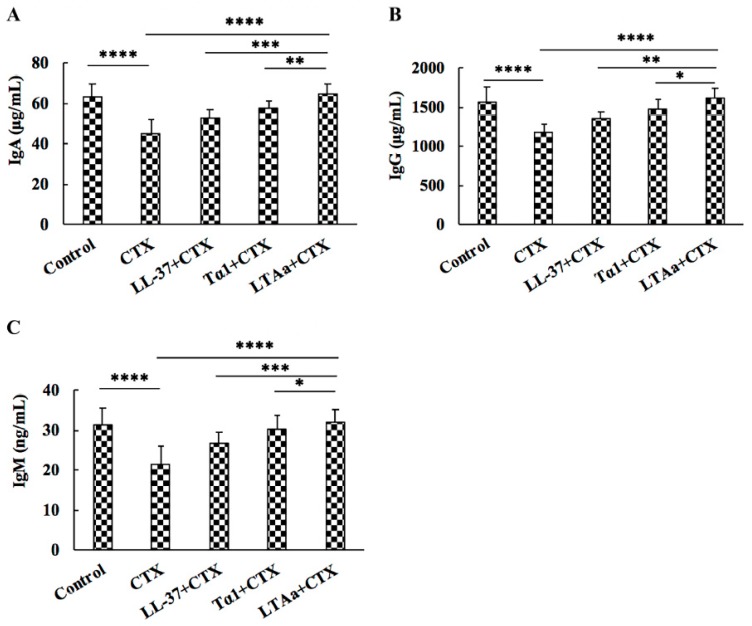
Effects of LTA_a_ on IgA (**A**), IgG (**B**), and IgM (**C**) levels in mice immunosuppressed by cyclophosphamide (CTX). Levels of IgA, IgG, and IgM were detected in serum from different groups of mice through ELISA. Data are means ± SEMs of 12 biological replicates. *, *p* ≤ 0.05; **, *p* ≤ 0.01; ***, *p* ≤ 0.001; and ****, *p* ≤ 0.0001.

To determine the effects of LTA_a_ on humoral immunity, IgA, IgG, and IgM levels in the sera of CTX-treated mice were evaluated by ELISA. As shown in Figure 10, serum IgA (Figure 10A), IgG (Figure 10B), and IgM (Figure 10C) levels were significantly decreased by CTX, and peptide-treated mice had significantly higher total serum IgA, IgG, and IgM levels than mice treated with CTX (Figure 10). Mice in the LTA_a_-treated group had similar IgA, IgG, and IgM levels as those in the control group.

### 2.9. Specific Binding of LTA_a_ to TLR4/MD-2

**Figure 11:**
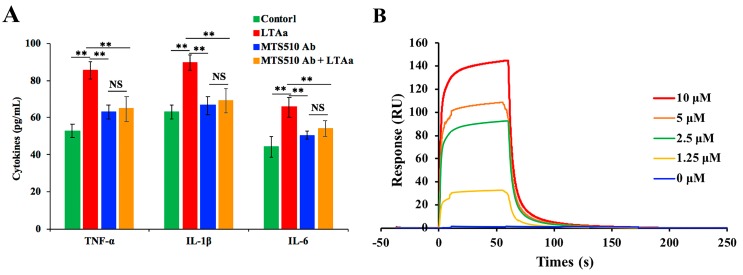
Binding of LTA_a_ to TLR4/MD-2. (**A**) RAW264.7 cells were incubated with phosphate-buffered saline or TLR4/MD-2 mAb (MTS510 Ab) for 1 h. The cells were then treated with or without 10 μg/mL peptides for 24 h. TNF-α, IL-6, and IL-1β levels in the cell culture supernatant were quantified by enzyme-linked immunosorbent assay. (**B**) The TLR4/MD2 complex was immobilized on a sensor chip, and LTA_a_ binding was analyzed by surface plasmon resonance. Data are means ± SEMs of three biological replicates. Not significant (NS), *p* > 0.05; and **, *p* ≤ 0.01.

In order to assess the binding of LTA_a_ to TLR4/MD-2, cells were incubated with PBS or TLR4/MD-2 mAb for 1 h. The cells were then treated with or without 10 μg/mL peptides for 24 h. TNF-α, IL-6, and IL-1β levels in the cell culture supernatant were quantified by ELISA. LTA_a_ caused a significant increase in the production of TNF-α, IL-6, and IL-1β (Figure 11A), and pretreatment with TLR4/MD-2 mAb significantly inhibited the TNF-α, IL-6, and IL-1β production induced by LTA_a_.

To confirm the binding of LTA_a_ to the TLR4/MD-2 complex, a SPR assay was run to analyze the binding kinetics of ligand-receptor interactions in detail (Figure 11B). Five different concentrations of LTA_a_ (0, 1.25, 2.5, 5, and 10 μM) were passed over immobilized TLR4/MD2. As shown in Figure 11B, LTA_a_ binding to the chip-bound protein exhibited a dose-dependent increase. The *K_a_* and *K_d_* values for LTA_a_ binding to TLR4/MD2 were 7.38 × 10^9^ s^−1^ and 3.62 × 10^−1^ M^−1^ s^−1^, respectively, and the *K_D_* was 4.91 × 10^–5^ μM.

### 2.10. LTA_a_ Activates the TLR4-NF-κB Pathway in CTX-Treated Mice

**Figure 12:**
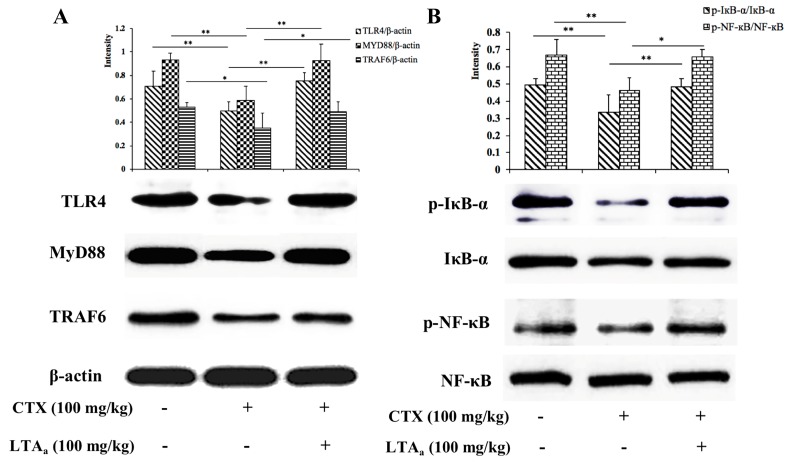
LTA_a_ activates the TLR4-NF-κB pathway. Phosphorylated and total protein levels of TLR4, MyD88, TRAF6, and β-actin (**A**). IκB-α and NF-κB (**B**) from serum were measured by western blot analysis. “+” means LTA_a_ or CTX addition, and “-” means without LTA_a_ or CTX addition. Data are means ± SEMs of three biological replicates. *, *p* ≤ 0.05; **, *p* ≤ 0.01.

We investigated the TLR4-NF-κB signaling pathway in CTX-treated mice treated with or without LTA_a_ to determine the mechanism underlying the immunomodulatory activity of LTA_a_. TLR4, MyD88, and TRAF6 expression levels in the CTX group were significantly lower than those in the control group (Figure 12A) or the LTA_a_-treated group. Compared to the CTX group, IκB-α and NF-κB phosphorylation significantly decreased in the LTA_a_-treated group (Figure 12B). These results suggest that one mechanism by which LTA_a_ modulates the immune system in mice is via the TLR4-NF-κB pathway.

## 3. Discussion

Immunity is a defensive response than can protect against disease by identifying and destroying harmful substances or microbiological toxins [1]. A suppressed immune system makes an organism more susceptible to infection, organ injury, and cancer [2], and immune function plays an important role in preventing and recovering from immune-mediated diseases. Therefore, it is necessary to develop a new immunomodulatory agent that can prevent and treat the diseases associated with immunosuppression.

Several factors, including work-related stress, pollution, and immunosuppressive agents, contribute to low immunity and poor health [29]. Immune response modulation is a crucial defense against disease [30]. In recent years, it has been reported that native immunomodulatory peptides have profound effects on the immune system, and have been regarded as ideal immunomodulatory candidates with a wide range of applications [31,32]. They have immune regulatory functions in cell proliferation, cytokine production, and macrophage phagocytosis [32,33,34]. LL-37 and Tα1 have shown enormous potential in the treatment of a range of immunosuppressive diseases [33,35], but possible cytotoxicity [23], low immunomodulatory activity according to peptide concentration, and weak physiological stability [9] reduce their clinical potential.

Hybridizing different immunomodulatory peptides is one of the most successful approaches in obtaining a novel immunomodulatory peptide with increased activity and physiological stability but decreased cytotoxicity [36,37]. In the present study, we designed three hybrid peptides comprising the Tα1 active center that included Tα1 (17–24), Tα1 (20–25), and Tα1 (20–27) with the core functional region of LL-37 (13–36).

TLR4/MD2 plays important roles in host defense by sensing conserved molecular patterns on microbial pathogens and mounting immune responses [4,5]. Therefore, targeting TLR4/MD2 is an important therapeutic strategy against immunosuppressive diseases. Initially, molecular docking was used to simply and effectively scan the binding mode of the immunomodulatory peptides. Of the three hybrid peptides, LTA_a_ had the most favorable docking scores to TLR4-MD-2. The immunomodulatory activity of the hybrid peptides was also assessed in RAW264.7 cells, which showed that LTAa exhibited higher immunomodulatory activity than the other peptides. Therefore, LTA_a_ was selected for a comprehensive analysis because it was the most active peptide.

A CCK-8 assay revealed that LTA_a_ had lower cytotoxicity than its parental peptides, possibly because of the hydrophobicity of the hybrid peptide, which was similar to that described in other studies [37].

As is the case for many peptide drugs, the half-life of Tα1 is short, which reduces its efficacy and bioavailability [33]. In order to maintain its clinical efficacy, it is necessary to administer repeat injections over a long treatment duration, so it is important to prolong the half-life of the peptide. Our results show that the in vitro half-life of LTA_a_ in rat plasma was prolonged to 4.2 h.

CTX can damage the structure of DNA, kill immune cells, interfere with the differentiation and proliferation of T and B cells, and decrease cellular and humoral immune responses [38,39,40], so we used CTX to immunosuppress the mice. Spleen and thymus indices are the most representative features of immune function and immune prognosis because these organs play important roles in nonspecific immunity [41]. Mice immunosuppressed by CTX had significantly lower body weights and spleen and thymus indices values than mice in the control group. However, post-CTX peptide treatment significantly reversed the decreases in body weight and spleen and thymus indices. Therefore, LTA_a_ was more potent than the parental peptides.

Macrophages are key participants in the innate immune response, and are the most rapid cell types to respond to invasion by pathogenic organisms [42]. Macrophage activation plays an important role in both innate and adaptive immunity [43]. Peritoneal macrophage phagocytosis decreased in CTX-treated mice, but post-treatment with LTA_a_ significantly increased it in comparison to the Tα1- and LL-37-pre-treated groups.

T lymphocytes are primary helper and effector cells in the adaptive immune response [44]. When the body is infected with pathogens or antigens, the CD4^+^:CD8^+^ ratio increases to release proinflammatory cytokines that help fight infections [45]. Consistently, we found that CTX decreased the CD4^+^:CD8^+^ ratio, and that LTA_a_ increased it. The LTA_a_-post-treated group had a higher CD4^+^:CD8^+^ ratio than the Tα1 or LL-37 groups, indicating that LTA_a_ improved immune function by regulating T lymphocyte subsets.

Cytokines play important roles in cell-cell communication in the immune system [46], and those such as TNF-α, IL-6, and IL-1β are involved in the preservation and restoration of homeostasis by coordinating lymphoid, inflammatory, and hematopoietic cells [41]. LTA_a_ significantly reversed the decrease in serum TNF-α, IL-6, and IL-1β levels caused by CTX. Despite the suppressive effect of the parental peptides on CTX-induced immunosuppression, the immunomodulatory activity of the parental peptides was lower than that of LTA_a_.

IgA, IgG, and IgM are important immunoglobulins that are involved in complement activation, opsonization, and toxin neutralization [47]. Consistent with the results of previous studies, IgA, IgM, and IgG levels had significantly decreased in CTX-treated mice, but post-treatment with LTA_a_ significantly reduced this effect. In addition, IgA, IgM, and IgG levels in the LTA_a_-post-treated group were significantly higher than in the Tα1- or LL-37-pre-treated groups.

Collectively, these results indicate that LTA_a_ has greater immunomodulatory potency and stability than its parental peptides, while also having minimal cytotoxicity. To identify the mechanisms underlying the observed immunomodulatory effects of LTA_a_ in CTX-treated mice, a comprehensive and detailed analysis was conducted.

We investigated whether the hybrid peptides exhibited immunomodulatory properties in RAW264.7 cells and in the mouse by binding to the TLR4-MD-2 complex as proposed. To investigate the ability of LTA_a_ to bind to the TLR4/MD-2 complex, binding assays were performed by ELISA. LTA_a_ caused a significant increase in TNF-α, IL-6, and IL-1β levels, but pre-treatment with TLR4/MD-2 mAb significantly inhibited the TNF-α, IL-6, and IL-1β production induced by LTA_a_. This suggests that LTA_a_ exhibits its immunomodulatory effects through the TLR4/MD-2 complex. The SPR results confirmed that LTA_a_ could effectively bind to the TLR4-MD2 complex, so LTA_a_ may depend upon binding to the TLR4/MD-2 complex for its immunomodulatory effects.

Nuclear factor-κB (NF-κB) is a crucial initial factor in host defenses that regulates inflammatory gene expression [48]. MyD88 is utilized by TLR4 and activates downstream NF-κB signaling. MyD88 recruits TRAF6, which then activates NF-κB signaling [49]. In the present study, the expression of the main proteins involved in the TLR4-NF-κB pathway was assessed in order to elucidate the immunomodulatory mechanism of LTA_a_. LTA_a_ activated the TLR4-NF-κB pathway by increasing the expression of TLR4, MyD88, and TRAF6 and the phosphorylation of IκB-α and NF-κB.

## 4. Materials and Methods

### 4.1. Hybrid Peptide Design

Three hybrid peptides, LTA_a_, LTA_b_, and LTA_c_, were designed by combining a characteristic fragment of LL-37 (the middle 13–36 residues) with the active center of Tα1_a_, Tα1_b_, or Tα1_c_. The amino acid sequences of the hybrid peptides and their parental peptides are listed in Table 1. The mean hydrophobicity of the peptides was calculated online using the bioinformatics program ProParam (ExPASy Proteomics Server: http://www.expasy.org/tools/protparam.html). Three-dimensional structures of the hybrid peptides LTAa, LTAb, and LTAc were built using I-TASSER (https://zhanglab.ccmb.med.umich.edu/I-TASSER/).

### 4.2. Peptide Synthesis

The hybrid peptides LTA_a_, LTA_b_, and LTA_c_ and their parental peptides LL-37 and Tα1 were synthesized and purified by KangLong Biochemistry (Jiangsu, China) using solid-phase methods. The purity of the peptides was determined to be greater than 95% by reverse-phase high-performance liquid chromatography (Sigma-Aldrich, Singapore) and mass spectrometry (MALDI-TOF MS, Model Autoflex, Bruker Daltonics Inc., Billerica, MA, USA) (Figure S1). The peptides were identified by mass spectrometry and dissolved in endotoxin-free water, before being stored at −80 °C.

### 4.3. Circular Dichroism Analysis

The secondary structures of hybrid peptides LTA_a_, LTA_b_, and LTA_c_ were determined by CD spectroscopy. The peptides were dissolved in sterile water and 25 mM sodium dodecyl sulfate (SDS) at a concentration of 0.1 mg/mL. The measurements were tested over a UV range of 190–250 nm using a Jasco-810 spectropolarimeter at 25 °C.

### 4.4. Hybrid Peptide Scan by Molecule Docking

The three-dimensional structures constructed for the hybrid peptides were then subjected to molecular docking. The initial structure of the TLR4/MD-2 complex was obtained from the Protein Data Bank (code: 2Z64). The initial TLR4/MD-2-hybrid peptide complex was produced by ZDOCK3.0.2. For each hybrid peptide, 3600 decoy structures were predicted by the rigid-body ZDOCK. The decoy with the lowest energy was chosen for the following flexible docking study. For each molecule, 1000 docking runs were performed by RosettaDock (version 3.5). The most plausible docking confirmation with the lowest interface energy was selected to scan the hybrid peptides.

### 4.5. Cell Culture

The murine macrophage cell line RAW264.7 was purchased from the Shanghai Cell Bank, Institute of Cell Biology, China Academy of Sciences (Shanghai, China) and maintained in Dulbecco’s modified Eagle’s medium (HyClone Logan, UT, USA) supplemented with 100 U/mL penicillin, 100 mg/mL streptomycin (Invitrogen), and 10% (v/v) fetal bovine serum (HyClone) at 37 °C in a humidified atmosphere (5% CO_2_, 95% air).

### 4.6. Cell Viability Assay

RAW264.7 viability was measured using a Cell Counting Kit-8 (CCK-8) Assay Kit (Dojindo) [50]. Briefly, cells were plated at a density of 1.0–2.0 × 10^4^ cells per well and treated with peptides (10–60 μg/mL) or without peptides. After incubation for 24 h or 72 h at 37 °C, the cells were incubated in CCK-8 solution for 4 h at 37 °C in darkness. The optical density (OD) was then measured using a microplate reader at 450 nm. Cell viability was calculated in the following way:

(1) $$\mathrm{cell}\;\mathrm{viablity}\;\left(\%\right)=\frac{\mathrm{OD}450\left(\mathrm{sample}\right)}{\mathrm{OD}450\left(\mathrm{control}\right)}\;\times 100\%$$

where OD450 (sample) is the absorbance at 450 nm by peptide-treated cells and OD450 (control) is the absorbance at 450 nm by the cells without peptides treated.

### 4.7. Immunomodulatory Activity in the RAW264.7 Cell Line

RAW264.7 cells were stimulated with or without 10 μg/mL peptides for 24 h at 37 °C in a humidified incubator with 5% CO_2_. Levels of TNF-α, IL-6, and IL-1β in the culture supernatant were measured using enzyme-linked immunosorbent assay (ELISA) kits according to the manufacturer’s instructions

### 4.8. Ex Vivo Stability of LTA_a_ in Plasma

The half-life of LL-37, Tα1, and LTA_a_ were determined in vitro following an incubation at 37 °C in rat plasma at different time points. The samples were collected into pre-chilled tubes containing 1 mL of acidic acetone (hydrochloric acid/acetone/H_2_O, 1:40:5, by volume). Subsequently, the mixture was centrifuged at 2 × 10^4^ rpm at 4 °C for 20 min. The precipitates were dried in vacuo. The dried precipitates were dissolved in 0.5 mL of 1 M acetic acid. The peptide analysis was carried out according to the protocol for the study of Tα1 using HPLC [51]. The half-lives of the target peptides were calculated by a logarithm-linear regression analysis of the peptide concentrations.

### 4.9. Animal Model

Female specific-pathogen-free (SPF) Balb/c mice (6–8 weeks of age, weighing 20 ± 2.0 g) were purchased from Charles River (Beijing, China). The mice were housed in a SPF environment at room temperature with a relative humidity of 55 ± 10% and allowed free access to food and water during the experiments. All of the animal protocols were approved by the Institutional Animal Care and Ethics Committee of the China Agricultural University (CAU20170430-4).

The mice were randomly divided into five groups (12 mice in each group): a control group, a cyclophosphamide (CTX; Sigma-Aldrich, St. Louis, MO, USA) group, a LL-37 group, a Tα1 group, and a LTA_a_ group. For the first 3 days, CTX (80 mg/kg mouse weight) was administered intraperitoneally once daily to establish the immunosuppressed animal model. From days 4 to 10 (7 days), peptides (10 mg/kg mouse weight) were administered intraperitoneally each day. The CTX group was only treated with CTX, and the control group was given sterile saline. The dose volume was 200 μL. Twenty-four hours after the last dose, the mice were killed and their tissues and blood collected for analysis. The body weight index was calculated in the following way:

(2) $$\mathrm{Index}\;\left(\%\right)=\frac{\mathrm{final}\;\mathrm{body}\;\mathrm{weight}-\mathrm{inital}\;\mathrm{body}\;\mathrm{weight}}{\mathrm{initial}\;\mathrm{body}\;\mathrm{weight}}\;\times 100\%$$

Spleen and thymus indices were calculated in the following way:

(3) $$\mathrm{Index}\;\left(\mathrm{mg}/\mathrm{kg}\right)=\frac{\mathrm{weight}\;\mathrm{of}\;\mathrm{thymus}\;\mathrm{or}\;\mathrm{spleen}}{\mathrm{body}\;\mathrm{weight}}$$

### 4.10. Preparation of Peritoneal Macrophages

Peritoneal macrophages were aseptically obtained from the different groups of mice by peritoneal lavage with 5 mL ice-cold phosphate-buffered saline (PBS). The peritoneal macrophages were further isolated by incubating peritoneal exudate cells at 37 °C in a moist atmosphere (5% CO_2_, 95% air) for 3 h to allow the cells to adhere. The adherent cells were washed twice and collected as peritoneal macrophages.

### 4.11. Peritoneal Macrophage Phagocytosis

The phagocytic capacity of the peritoneal macrophages was measured by the neutral red uptake method [52]. The peritoneal macrophages were seeded in a 96-well plate and incubated at 37 °C in a moist atmosphere (5% CO_2_, 95% air). A neutral red solution (50 mg/mL) was added to each well and incubated for an additional 3 h. The cells were then washed with PBS three times and the phagocytotic dye was extracted with 100 μL 1% acetic acid solution (v/v) in 50% ethanol (v/v). The OD was measured using a microplate reader at 540 nm. The phagocytic rate was calculated in the following way:

(4) $$\mathrm{hagocytic}\;\mathrm{rate}\;\left(\%\right)=\frac{\mathrm{OD}540\left(\mathrm{sample}\right)}{\mathrm{OD}540\left(\mathrm{control}\right)}\;\times 100\%$$

where OD540 (sample) is the absorbance at 540 nm by peptide-treated cells and OD540 (control) is the absorbance at 540 nm by the cells without peptides treated.

### 4.12. Flow Cytometry

The spleen was collected, ground, and passed through 40-μm-mesh cell strainers filled with PBS to harvest the cell suspension. The cells were stained with CD3- PerCP, CD4-APC, and CD8-FITC for 30 min at 4 °C for T lymphocyte measurements. The results were analyzed using a Guava^®^ easyCyte^TM^ 6-21 system and Guavasoft 3.1.1 software (Millipore, Burlington, MA, USA).

### 4.13. Serum Cytokine and Immunoglobulin (Ig) Measurements by ELISA

Serum was obtained from blood by centrifugation at 1000 × g for 20 min. Levels of TNF-α, IL-6, IL-1β, IgG, IgA, and IgM in the serum were detected by ELISA.

### 4.14. Binding Assay of LTA_a_ to TLR4/MD-2

RAW264.7 cells were treated with PBS or an anti-mouse mAbTLR4/MD-2 complex (MTS510 Ab) (eBioscience, San Diego, CA, USA) at 37 °C for 1 h, and RAW264.7 cells were treated with or without 10 μg/mL peptides for 24 h at 37 °C in a moist atmosphere (5% CO2, 95% air). Levels of TNF-α, IL-6, and IL-1β in the culture supernatant were detected by ELISA.

Surface plasmon resonance (SPR) experiments were performed using a ProteOn^TM^ XPR36 instrument (Bio-Rad, Hercules, CA, USA) with a ProteOn^TM^ GLH sensor chip (Bio-Rad) at 25 °C. PBS supplemented with 0.1% Tween 20 (containing 2% dimethyl sulfoxide) was used as a running buffer and was continuously passed into the reaction chamber at 30 μL/min. A SPR sensing chip with recombinant TLR4/MD2 (R&D Systems, Minneapolis, MN, USA) that was immobilized by amino coupling was used to capture the peptide, according to the manufacturer’s protocol. Varying concentrations of the peptide (0, 1.25, 2.5, 5, and 10 mM) were injected into the chip to check for binding with TLR4/MD2. The running buffer was injected into the empty channel as a reference. Sensor chip regeneration and desorption were achieved by injecting 10 mM Gly-HCl buffer (pH 2.5) before the next round of analyses. The experimental data were analyzed by ProteOn Manager^TM^ software (version 2.0). Binding curves were processed for the initial injection alignment and baseline, and a reference-subtracted sensorgram was globally fitted to the curves to describe a homogeneous 1:1 model. Data from the protein surfaces were grouped together to fit the kinetic rate constants (*K_a_* and *Kd*). The equilibrium dissociation constant *(K_D_*) for the peptide-TLR4/MD2 interaction was calculated using the following equation:

(5) $$\mathrm{KD}=\frac{\mathrm{Kd}}{\mathrm{Ka}}$$

### 4.15. Western Blot Analysis

Whole protein in the serum was collected using a whole-protein extraction kit (Nanjing KeyGEN Biotech, Nanjing, China) according to the manufacturer’s instructions. The protein concentration was measured using a bicinchoninic acid kit (Nanjing KeyGEN Biotech). The different proteins were separated by sodium dodecyl sulfate polyacrylamide gel electrophoresis and transferred onto polyvinylidene difluoride membranes (Bio-Rad). The membranes were blocked in Tris-buffered saline and Tween 20 (TBST) blocking buffer that contained 5% nonfat dried milk for 2 h at room temperature and immunoblotted with primary specific antibodies (anti-TLR4, anti-TRAF6, anti-TAK1, IκB-α, p-IκB-α, anti-NF-κB (p65), anti-p-NF-κB (p-p65), and anti-β-actin (Abcam, UK). After washing with TBST, the strips were incubated with horse-radish peroxidase-conjugated goat anti-mouse IgG (HuaAn, Hangzhou, China). Proteins were detected using a SuperSignal™ West Femto Maximum Sensitivity Substrate (Thermofisher) and visualized using a ChemiDoc™ MP Imaging System (Bio-Rad).

### 4.16. Statistics

All data are expressed as the means ± SEMs of at least three independent experiments. Statistical comparisons were performed by Student’s t test and analysis of variance using GraphPad Prism v6 software. Significance was set as follows: not significant (NS), *p* > 0.05; *, *p* ≤ 0.05; **, *p* ≤ 0.01; ***, *p* ≤ 0.001; and ****, *p* ≤ 0.0001.

## 5. Conclusions

**Figure 13:**
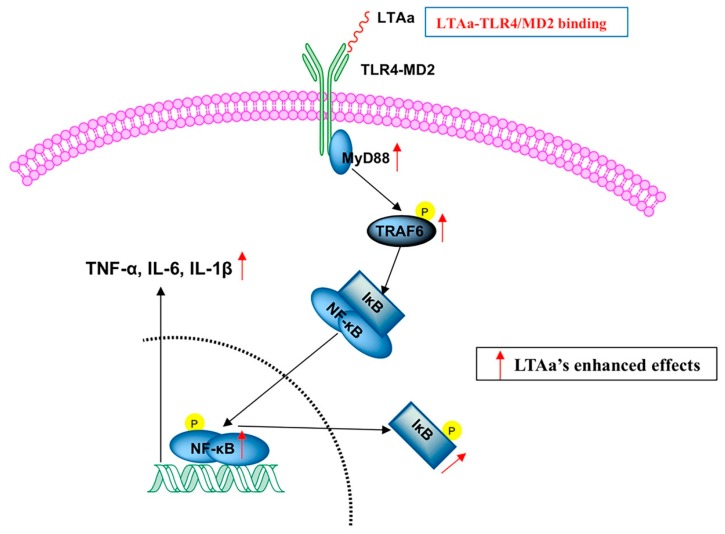
A sketch map of immunoregulatory induced by LTA_a_.

In this study, a feasible approach for the design of new immunomodulatory peptides by hybridizing different peptides was proposed (Figure 13). A novel hybrid peptide (LTA_a_) with high immunomodulatory activity and stability and minimal cytotoxicity was identified by molecule docking and in vitro experiments. Our study confirmed the immunomodulatory effects of LTA_a_ using a CTX-immunosuppressed murine model. LTA_a_ reversed immunosuppression, increased immune organ function, activated peritoneal macrophage phagocytosis, regulated T lymphocyte subsets, and increased cytokine (IL-6, TNF-α, and IL-1β) and Ig (IgA, IgG, and IgM) contents. The immunomodulatory effects of LTAa were mainly caused by its binding to the TLR4/MD-2 complex and activation of the NF-κB signaling pathway. Overall, our findings suggest that LTA_a_ can be developed as a novel immunostimulant for the food and pharmaceutical industries.

## Figures and Tables

**Table 1:** Design and key physicochemical parameters of the peptides.

Peptide	Sequence	H ^a^	Net Charge
LL-37	LLGDFFRKSKEKIGKEFKRIVQRIKDFLRNLVPRTES	−0.559	+6
Tα1	SDAAVDTSSEITTKDLKEKKEVVEEAEN	−1.029	−5
LTA_a_	IGKEFKRIVQRIKDFLRNLVPRTEKEKKEVVE	−0.894	+4
LTA_b_	IGKEFKRIVQRIKDFLRNLVPRTEEVVEEA	−0.503	+1
LTA_c_	IGKEFKRIVQRIKDFLRNLVPRTEEVVEEAEN	−0.691	0

^a^ The mean hydrophobicity (H) was the total hydrophobicity (sum of all residue hydrophobicity indices) divided by the number of residues.

**Table 2:** Half-life of LTA_a_ in plasma.

Peptide	LL-37	Tα1	LTA_a_
**t_1/2_ (h)**	3.1 ± 0.87 ^b^	1.8 ± 0.24 ^c^	4.2 ± 1.13 ^a^

Data are means ± SEMs of three biological replicates. Means with different superscript letters within the same row significantly differed (*p* < 0.01).

## Supplementary Materials

Supplementary materials can be found at https://www.mdpi.com/1422-0067/20/24/6161/s1.
