# Supplementary figures and images for: Development of a Highly Efficient Hybrid Peptide That Increases Immunomodulatory Activity Via the TLR4-Mediated Nuclear Factor-κB Signaling Pathway
Source: Int J Mol Sci. 2019 Dec 6;20(24):6161. doi: 10.3390/ijms20246161 (PMC6940896; doi:10.3390/ijms20246161)

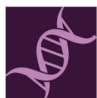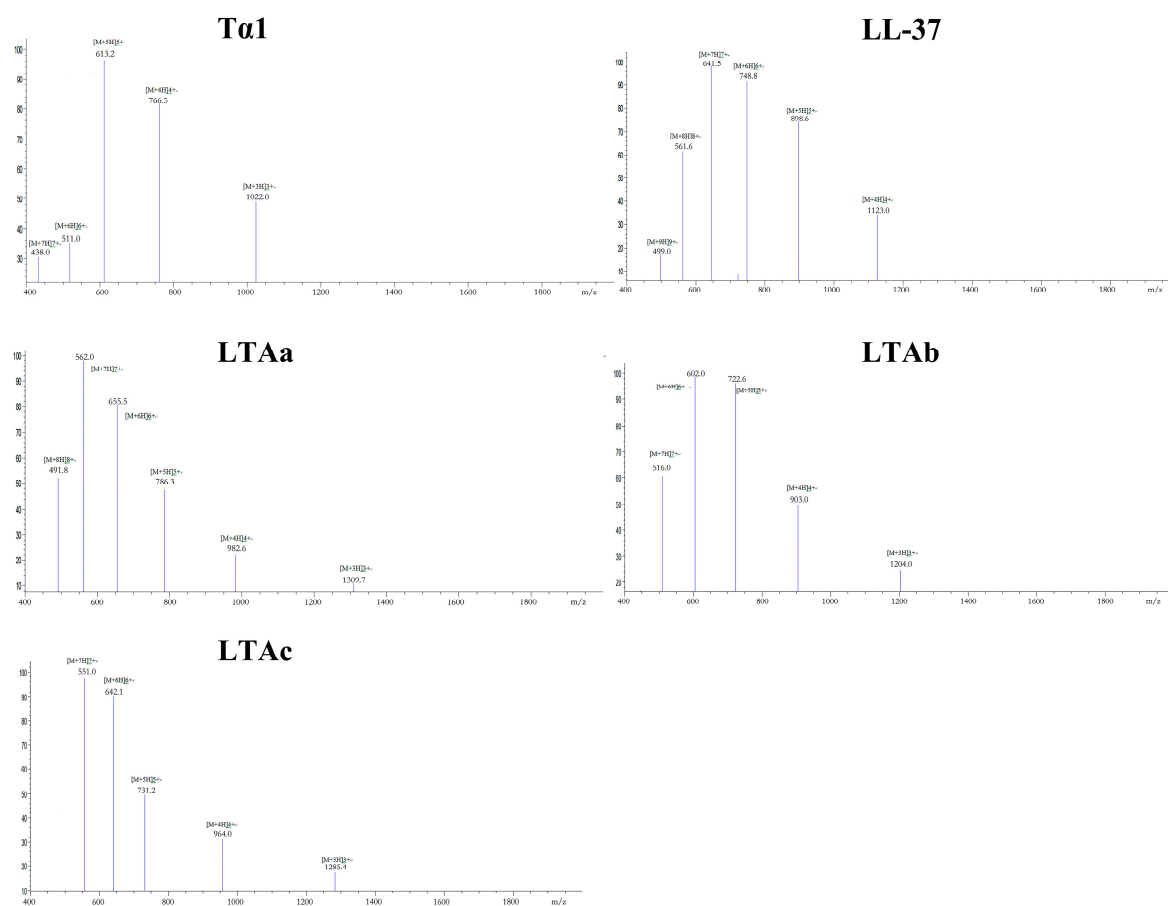

**Figure S1.** Mass spectrometry analysis of purity peptides.

Supplement: Supplementary file 1 [file ijms-20-06161-s001.pdf]
